# Double-probe rolling circle amplification with lateral flow readout for highly specific detection of the phytopathogenic and keratocutaneous pathogen *Fusarium proliferatum*

**DOI:** 10.3389/fmicb.2026.1991703

**Published:** 2026-10-28

**Authors:** Fuguo Liu, Wei Xia, Hongsen Wu, Yueyao Wang, Yu Zhang, Xiaofang Li

**Affiliations:** 1 Jia Sixie College of Agriculture, Weifang University of Science and Technology, Weifang, China; 2 Shouguang Vegetable Hi-Tech Demonstration Park Management Service Center, Weifang, China

**Keywords:** dpRCA–LFA, dual-probe recognition, *F. Proliferatum*, pathogen, rapid detection

## Abstract

*Fusarium proliferatum* is a soilborne phytopathogenic fungus that poses a serious threat to global crop production owing to its broad host range, strong toxigenicity, and wide distribution. Beyond its agricultural impact, this fungus has also emerged as an opportunistic pathogen capable of infecting human cornea and skin, particularly in immunocompromised individuals. Rapid and accurate on-site detection is critical for early disease intervention. In this study, we adapted and optimized a dual-probe rolling circle amplification integrated with lateral flow assay (dpRCA-LFA) for the specific detection of *F. proliferatum*, building on previously reported dpRCA-LFA principles. The main contributions of this work are the design of *F. proliferatum*-specific probes and the optimization of the reaction system for soil and field sample testing. The conventional single padlock probe was redesigned into two probes (long probe and short probe), establishing a dual-recognition mechanism that substantially enhances specificity. Under optimized conditions, the lowest concentration at which a visible positive signal was observed was 6.6 × 10^−4^ ng μL^−1^ for genomic DNA and 2.7 × 10^2^ copies μL^−1^ for recombinant plasmid containing the TEF-1α. These values represent the detection end-points under the tested conditions rather than statistically established limits of detection, and correspond to a 100-fold improvement over conventional PCR-AGE in the tested dilution series. Specificity testing against 19 fungal strains yielded positive signals exclusively for *F. proliferatum*. The assay exhibited robust anti-interference performance, with consistent detection even when target DNA constituted only 1% of total DNA. In artificially inoculated soil samples, dpRCA–LFA achieved a 10-fold higher sensitivity than PCR–AGE. In field samples, dpRCA-LFA identified five positive samples versus four detected by PCR-AGE. Given the limited number of field samples, this difference is presented as a preliminary observation rather than a demonstration of superior field performance. The entire process, from amplification to visual result interpretation, can be completed within 90 min without requiring specialized equipment. Collectively, dpRCA-LFA offers a rapid, sensitive, and specific tool for the detection of *F. proliferatum* in fungal cultures and soil matrices, with potential utility for decentralized screening after suitable DNA extraction and controlled reaction processing. Clinical application remains a future direction.

## 1 Introduction

*Fusarium proliferatum*, a species belonging to the *Fusarium*, initially produces white colonies that gradually transition to light pink and eventually to nearly black, with concentric ring patterns and woolly, curled aerial mycelia. As a widespread agricultural phytopathogen, this fungus has a broad host range and can infect a variety of economically important major crops, including maize, wheat, millet, and legumes, causing multiple plant diseases and resulting in severe economic losses (Cendoya et al., 2018; Ekwomadu and Mwanza, 2023). In addition to its agricultural and mycotoxigenic risks, *F. proliferatum* has been increasingly documented as a causative agent of human infections, with case reports confirming its involvement in keratitis (corneal infections) and cutaneous (skin) lesions, particularly following traumatic inoculation or in patients with underlying immunodeficiency (Su et al., 2016; Sun et al., 2019). This dual-pathogenic nature—affecting both plants and humans—elevates *F. proliferatum* from a purely agricultural concern to a broader public health issue. However, the present study focuses on the development and validation of the dpRCA-LFA assay for detection of *F. proliferatum* in fungal cultures and soil matrices, and does not include clinical validation. Clinical application of this assay remains a future research direction.

To reduce the potential economic losses caused by plant diseases, it is imperative to enhance monitoring of pathogens and prevent the occurrence of disease outbreaks. Morphological identification, a traditional method for fungal taxonomy, is commonly employed and primarily involves observation of colony characteristics and spore morphology (Zhang et al., 2023). Although the method is widely applicable and allows rapid detection, it may be prone to errors due to morphological similarities among cultures or variations in phenotypic traits under different culture conditions (e.g., medium type, temperature) (Harish et al., 2023; Ma et al., 2023). Therefore, morphological identification can be subject to inaccuracies. Enzyme-linked immunosorbent assay (ELISA), as a representative immunological detection technique, enables accurate fungal detection. However, the preparation of antibodies is complex and costly, and cross-reactivity among closely related genus may produce false-positive results (Attaluri and Dharavath, 2023; Charoenvilaisiri et al., 2021). Conventional molecular detection methods, such as polymerase chain reaction (PCR), quantitative real-time PCR (qPCR), and fluorescence *in situ* hybridization, have overcome the limitations of traditional morphology-based techniques and significantly improved detection accuracy. Nevertheless, these methods require sophisticated and expensive equipment (e.g., real-time PCR thermal cyclers, fluorescence microscopes) and involve laborious procedures, making them unsuitable for on-site detection (Venbrux et al., 2023; Zhang et al., 2025).

Isothermal amplification techniques, represented by loop–mediated isothermal amplification (LAMP), recombinase polymerase amplification (RPA), and rolling circle amplification (RCA), do not require specialized instrumentation (Ivanov et al., 2021; Qin et al., 2019). Nevertheless, LAMP necessitates the design of 4–6 specific primers targeting 6–8 regions of the target gene, rendering primer design challenging and thereby introducing assay uncertainty (Muthukumar et al., 2025). The core enzymes used in RPA—including recombinase, single-stranded DNA-binding proteins, and polymerase—along with its reaction buffer, are typically protected by patents, resulting in a unit cost substantially higher than that of PCR or LAMP reagents. Moreover, the method requires longer primers (approximately 30–35 nt), and the success rate of primer design is lower than that of conventional PCR (Bhat et al., 2022). In comparison, RCA requires only 1–2 primers, far fewer than LAMP, and its reagents are more affordable than those required for RPA, making it more suitable for rapid on-site detection.

RCA is an isothermal amplification technique that relies primarily on a padlock probe (PLP), which consists of two components: a target-recognition region (binding to the target) and a linker region (primer and hybridization probe) (Liu et al., 2025; Nie et al., 2017). The linear PLP can be circularized under the catalysis of a ligase only when the target-binding region of the PLP is perfectly complementary to the template DNA. Subsequently, the circularized PLP is continuously replicated under the action of a polymerase and primers. However, it has been reported that PLP probes can also undergo circularization even in the absence of exact complementary pairing with the template, giving rise to false-positive results (Liu et al., 2019). To address this issue, the present study splits the conventional single PLP into a long probe (LP) and a short probe (SP), adapting the previously reported dual-probe rolling circle amplification (dpRCA) concept for the detection of *F. proliferatum*.

The lateral flow assay (LFA), a paper chromatography-based biosensor, offers the advantages of simple operation, rapidity, high sensitivity, low cost, and ease of result interpretation, and has thus been widely employed in diverse detection fields (Qin et al., 2020). During LFA detection, the biotin-labeled amplification products are hybridized with probes labeled with 5-Carboxyfluorescein (FAM), generating positive products that bearing both biotin and FAM. Subsequently, the positive product is dispensed onto the sample pad and migrates slowly along with the chromatographic running buffer via capillary action (Figure 1). Upon reaching the colloidal gold pad, the product with FAM binds to colloidal gold-labeled anti-FAM antibodies, forming a “biotin–FAM–FAM antibody–colloidal gold” complex. As the complex continues to migrate to the test line, it binds to anti-biotin antibodies immobilized on the membrane, forming a “biotin antibody–biotin–FAM–FAM antibody–colloidal gold” complex. At this stage, the colloidal gold particles aggregate at the test line, producing a visible red line. In the absence of amplification products, the anti-FAM antibodies on the colloidal gold pad pass through the test line and bind to the anti-anti-FAM antibodies immobilized on the control line, where the aggregation of colloidal gold particles also produces a red line. The dpRCA products, when subjected to the aforementioned LFA analysis, enable the determination of the presence or absence of amplification products simply by assessing the coloration of the test line, with the entire procedure completed within 10 min.

**Figure 1:**
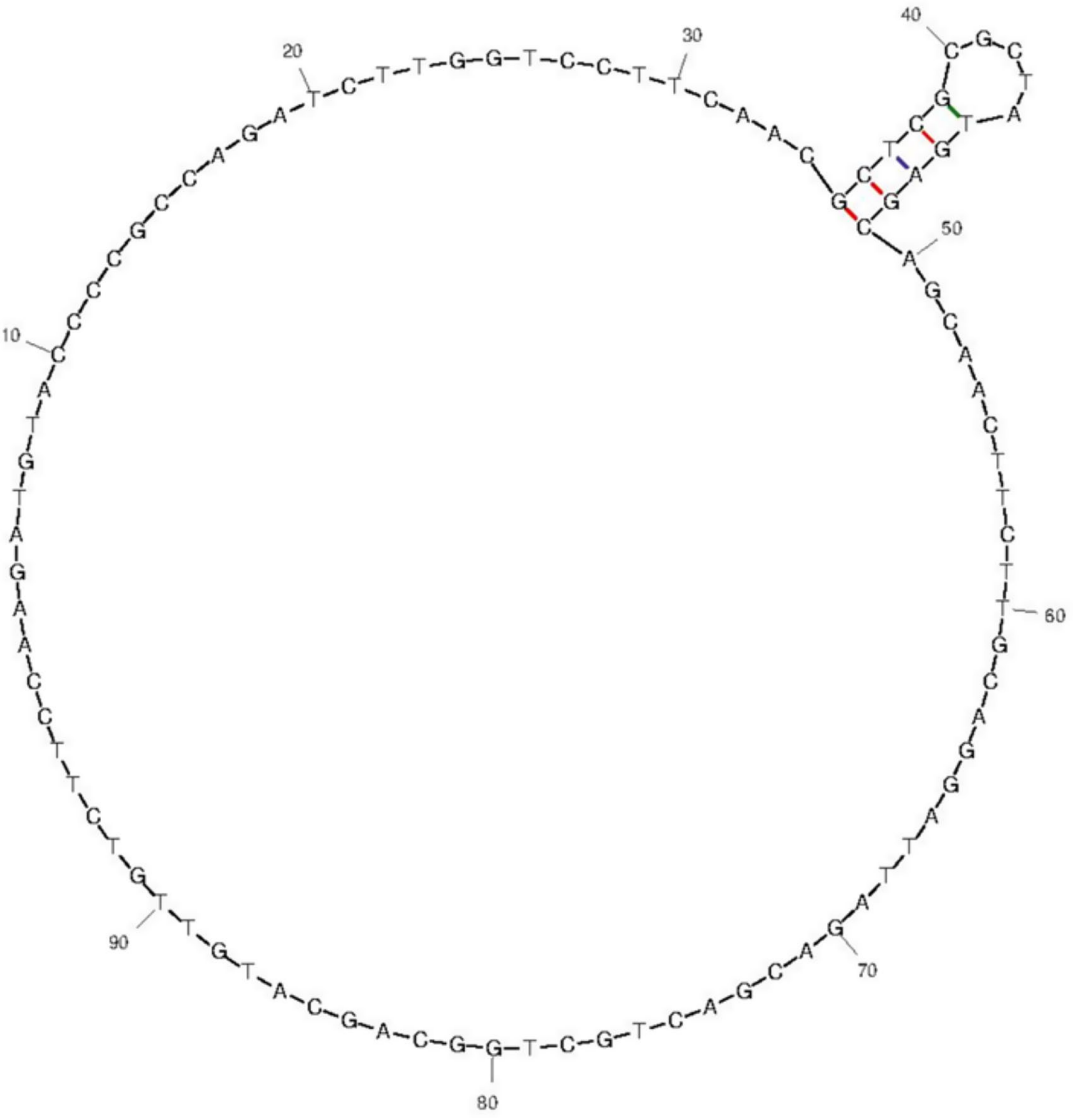
Predicted secondary structure of the LP and SP.

This study describes the adaptation and optimization of a rapid identification assay for *F. proliferatum*, designated as dpRCA-LFA, which couples dpRCA-mediated specific amplification with LFA-based visual detection, building on previously reported dpRCA-LFA principles (Qin et al., 2020; Tian et al., 2025). First, probes and specific primers were designed targeting the translation elongation factor 1-alpha (TEF-1α) gene of *F. proliferatum*, the actin gene of *Xenopus laevis*, and the actin gene of *Azumapecten farreri*, respectively. Following the initial setup of a dpRCA-agarose gel electrophoresis (AGE), we optimized critical reaction conditions, including probe concentration, ligation time, ligation temperature, amplification time, and amplification temperature. Under the optimal reaction conditions, common fungal species were selected to evaluate the specificity and anti-interference performance of the developed method. The detection sensitivity of dpRCA-AGE/dpRCA-LFA was assessed using both genomic DNA and recombinant plasmid DNA containing the TEF-1α gene. Furthermore, the practical applicability of the method was assessed by testing simulated and field-collected samples, with a comparative analysis performed between dpRCA-AGE/dpRCA-LFA and PCR-AGE.

## 2 Materials and methods

### 2.1 Tested strains and culture conditions

The strains used in this study are listed in Table 1. All strains were cultured on potato dextrose agar (PDA) medium. The PDA medium (1 L) was prepared with the following composition: potato extract powder 10.0 g, glucose 20.0 g, KH₂PO₄ 3.0 g, MgSO₄·7H₂O 1.5 g, thiamine 0.008 g, and agar 1.5–2.0 g. The culture conditions were 25 °C for 5–7 days.

**Table 1:** List of strains and results of dpRCA–LFA.

Strains	dpRCA–LFA
*Fusarium proliferatum*	+
*Fusarium andiyazi*	−
*Fusarium asiaticum*	−
*Fusarium avenaceum*	−
*Fusarium boothii*	−
*Fusarium concentricum*	−
*Fusarium culmorum*	−
*Fusarium equiseti*	−
*Fusarium fujikuroi*	−
*Fusarium incarnatum*	−
*Fusarium graminearum*	−
*Fusarium meridionale*	−
*Fusarium moniliforme*	−
*Fusarium oxysporum*	−
*Fusarium pseudograminearum*	−
*Fusarium solani*	−
*Fusarium temperatum*	−
*Fusarium verticillioides*	−
*Fusarium tricinctum*	−

### 2.2 DNA extraction, PCR amplification, and sequencing

The cultures of the tested strains (including both target and control strains) grown for 5–7 days were filtered to collect the mycelial pellets. Genomic DNA was extracted from the collected mycelial pellets using the Ezup Column Fungi Genomic DNA Purification Kit (Sangon Biotech, Shanghai, China). The purity of DNA extracted from fungal cultures was measured using a UV spectrophotometer by the absorbance ratio at 260 nm and 280 nm (A260/A280), and the samples were analyzed by 1% AGE. These quality checks were applied only to DNA extracted from fungal cultures for target sequence confirmation. For soil and field samples, DNA was extracted using the corresponding kits according to the manufacturer’s instructions and used directly in the assay without additional spectrophotometric or electrophoretic quality assessment. DNA samples were kept at −20 °C. Next, using the genomic DNA as template, the TEF-1α sequences were amplified with primers TEF1/TEF2 (O'Donnell and Cigelnik, 1997). The PCR products were analyzed by 2.0% AGE and then sequenced at Sangon Biotech (Shanghai) Co., Ltd.

### 2.3 Design of primers and probes

The sequences obtained from sequencing were trimmed to remove the primer sequences (TEF1/TEF2). The resulting sequences were then submitted to the NCBI website^1^ and analyzed using the online BLASTn tool to confirm the authenticity of the target sequences. The TEF-1α of related species (the strains belong to genus *Fusarium*) available in Genebank were downloaded (Table 2). Together with the sequencing results obtained in this study, a ClustalX Multiple Alignment analysis was performed using BioEdit software to visually identify the hypervariable regions of *F. proliferatum*, which were subsequently used as the terminal regions (T1 and T2) of the long probe (LP) and the short probe (SP), respectively. Using the actin gene of *Xenopus laevis* (Accession No. NM_001086982.1), a species phylogenetically distant from the target fungus, as the reference sequence, the amplification primers P1/P2 were designed with the assistance of the Primer Premier 5.0. Meanwhile, based on the actin gene of *Azumapecten farreri* (Accession No. KJ081194.1), the hybridization probe (HP) (18–24 nt) was designed using Primer Premier 5.0. The probes were ultimately arranged in the order of LP (T1–HP–P1c–P2–T2) + SP. The secondary structure of the circularized probe was analyzed using the UNAFold tool^2^. If necessary, the base sequences of the primers (P1/P2) and the HP region were adjusted to avoid excessive secondary structure (Figure 1). Additionally, specific primers FpF/FpR for *F. proliferatum* were designed based on the hypervariable regions of the target sequence. All probes and primers designed in the study (Table 3) were synthesized by Sangon Biotech (Shanghai) Co., Ltd.

**Table 2:** List of homolog strain introduced into the design of probe, with GenBank accession numbers of their TEF-1α sequences.

Strain	Accession no.
*Fusarium proliferatum*	KC121066.1
*Fusarium pseudograminearum*	PV907194.1
*Fusarium avenaceum*	MK111429.1
*Fusarium oxysporum*	MT738958.1
*Fusarium clavum*	PZ094653.1
*Gibberella intermedia*	JF270233.1
*Fusarium fujikuroi*	PX939546.1
*Fusarium verticillioides*	PX939541.1
*Fusarium tricinctum*	PX939518.1
*Fusarium temperatum*	KJ137018.1

**Table 3:** Design results of primer/probe.

Probe/primer	Sequence (5′-3′)	Length	Reaction
LP^a^	CGCCAGATCTTGGTCcttcaacgctcgcgctatgagcagcaacttcttgcaggattagacgactgctggCAGCATGTTG	79	dpRCA/dpRCA–LFA
SP^b^	TCTTCCAAGATGTACCC	17	dpRCA/dpRCA–LFA
PLP	CGCCAGATCTTGGTCCTTCAACGCTCGCGCTATGAGCAGCAACTTCTTGCAGGATTAGACGACTGCTGGCAGCATGTTGTCTTCCAAGATGTACCC	96	RCA
P1^c^	CCAGCAGTCGTCTAATCC	18	dpRCA/dpRCA–LFA
P2	GAGCAGCAACTTCTTGCA	18	dpRCA/dpRCA-LFA
HP^d^	CTTCAACGCTCGCGCTAT	18	dpRCA–LFA
FPR	GCGTTTTTGCCCTTTCCTGT	20	PCR
FPF	AACCCAGGCGTACTTGAAGG	20	PCR
TEF1	AACATGCGTGAGATTGTAAGT	21	PCR
TEF2	TAGTGACCCTTGGCCCAGTTG	21	PCR

^a^The lowercase letters denote the internal sequence linking both ends, while the capital letters denote both terminal regions recognizing target sequence. The probe LP is labeled with phosphoric acid at the 5′ end.

^b^The probe SP is labeled with phosphoric acid at the 5′ end.

^c^The primer P1 is labeled with biotin at the 5′ end.

^d^The probe HP is labeled with FAM at the 5′ end.

### 2.4 Establishment of PCR, dpRCA–AGE and dpRCA–LFA

The PCR system was established with the following components: 1 × PCR buffer (Vazyme, Nanjing), 0.4 μM of each primer, and 10 ng of template DNA. The amplification program was as follows: initial denaturation at 94 °C for 3 min; followed by 29 cycles of denaturation at 95 °C for 15 s, annealing at 55 °C for 15 s, and extension at 72 °C for 15 s; and a final extension step at 72 °C for 5 min.

The dpRCA consisted of two stages: circular ligation of the probe (LP and SP), and amplification of the cyclized probe. (1) Circular ligation of the probe. The ligation mixture contained 1 × ligase buffer, 2 nM of LP, 2 nM of SP, 40 U μL^−1^ of T4 DNA ligase (Vazyme, Nanjing), and template DNA (approximately 10 ng). The ligation program was as follows: pre-denaturation at 95 °C for 4 min; 6 cycles of 95 °C for 30 s and 55 °C for 3 min; followed by incubation at 95 °C for 10 min, and immediate ice bath for 5 min to disrupt secondary structures. (2) Amplification of the cyclized probe. The amplification mixture contained 4 U Bst DNA polymerase, 1 × Bst DNA polymerase buffer, 6 mM of MgSO_4_, 1.4 mM of dNTP mixture, 0.4 μM of each primer, and 2 μL of the ligation product. The amplification program was carried out at 62 °C for 30 min. The dpRCA products were analyzed by 2% AGE.

Under the optimized dpRCA reaction system, the original primer P1 was replaced with biotin-labeled primer P1, and a FAM-labeled HP was simultaneously introduced. The amplification of the cyclized probe was then performed as described in Section “2.4. Establishment of PCR, dpRCA–AGE and dpRCA–LFA”. After the reaction, 8 μL of the reaction mixture was thoroughly mixed with 92 μL of LFA buffer, followed by dipping an LFA into the mixture for approximately 5–10 min. Results were read visually at 30 min after sample application under ambient room lighting with the strips placed on a A4-size paper. A sample was scored as positive when a distinct pink/red test line was visible together with a visible control line; a sample was scored as negative when only the control line was visible; and a sample was considered invalid when the control line was absent, in which case the sample was re-tested. Strips were documented under the same lighting conditions using smartphone; images were used for documentation only and were not used for standardized intensity quantification. Each treatment was performed in triplicate.

### 2.5 Specificity of the dpRCA–LFA

To validate the specificity of the established dpRCA–LFA, a total of 19 representative strains (including *F. proliferatum*) were selected as test strains. The experimental procedure was as follows. PCR was performed using the genomic DNA of each test strain as template with the universal primers TEF1/TEF2 to confirm whether the template quality was suitable for subsequent experiment. Then, RCA and dpRCA assays were conducted using the same templates under the optimized conditions. The products from both PCR, RCA and dpRCA were analyzed by 2% AGE. In addition, the dpRCA products were further subjected to LFA, and the results were interpreted based on the visual inspection of band development on the test lines and control lines. Each treatment was performed in triplicate.

### 2.6 Sensitivity of the dpRCA–LFA

The sensitivity of the dpRCA–LFA was evaluated using genomic DNA and the recombinant plasmid containing the TEF-1α. The genomic DNA was serially diluted 10-fold with nuclease-free water to prepare six concentration ranging from 6.6 × 10^0^ ng μL^−1^ to 6.6 × 10^−5^ ng μL^−1^. Similarly, the concentration of the recombinant plasmid containing the TEF-1α sequence of *F. proliferatum* was measured using a UV spectrophotometer, and the copy number of the plasmid concentration (CNPC) was calculated according to the following formula: CNPC = (C × 10^−9^ × 6.02 × 10^23^)/(N × 660), where C and N represent the plasmid concentration (ng μL^−1^) and the base number of the recombinant plasmid, respectively. The recombinant plasmid DNA was serially diluted 10-fold with nuclease-free water to prepare six concentration gradients, ranging from 2.7 × 10^6^ copies μL^−1^ to 2.7 × 10^1^ copies μL^−1^. PCR–AGE, dpRCA–AGE, and dpRCA–LFA were performed using the above-prepared test samples as templates in parallel, comparing the sensitivities of the two methods. Each treatment was performed in triplicate. The reported detection values represent the lowest concentration at which a visible positive signal was observed in the serial dilution experiments, i.e., the end-point of detection under the tested conditions. They should be distinguished from a statistically defined analytical limit of detection (LOD), which would require replicated low-concentration measurements.

### 2.7 Assessment of anti-interference capability of dpRCA–LFA

The specificity and stability of the dpRCA–LFA assay in complex samples were further assessed through interference experiment. Genomic DNA of the interfering strain (*F. graminearum*) and the target strain (*F. proliferatum*) were mixed at various mass ratios (100,1, 10,1, 1,1, 1,10, and 1:100). The resulting DNA mixtures were then analyzed in parallel by conventional PCR–AGE, dpRCA–AGE, and dpRCA–LFA, respectively. Each treatment was performed in triplicate.

### 2.8 Recovery assay in spiked soil samples

Soil samples were sterilized by autoclaving at 121 °C for 20 min, and the absence of *F. proliferatum* was verified prior to use. Meanwhile, a laboratory-preserved strain of *F. proliferatum* was inoculated onto PDA medium and cultured for several days to induce sporulation. The spores were then harvested by adding an appropriate volume of sterile water containing 0.005% Tween 80 to the culture plates, and the resulting spore suspension was inoculated into the sterilized soil, followed by incubation for 7 days. Subsequently, 1 g of the treated soil sample was thoroughly mixed with 1 mL of sterile water, allowed to stand for 30 min, and centrifuged at 3000 rpm for 5 min. The supernatant was collected, and genomic DNA was extracted using a Fungal Genomic DNA Rapid Extraction Kit (Sangon Biotech, Shanghai, China) according to the instructions of manufacturer. The extracted genomic DNA was then serially 10-fold diluted with nuclease-free water to obtain six concentration gradients ranging from 1.3 × 10^2^ to 1.3 × 10^−3^ ng μL^−1^. Finally, using the above-prepared DNA samples and nuclease-free water as templates, conventional PCR–AGE, dpRCA–AGE, and dpRCA–LFA were performed in parallel. Each treatment was performed in triplicate.

### 2.9 Detection of field samples

In August 2024, a total of eight field soil samples were collected from Weifang, Zibo, Jinan, and Binzhou, Shandong Province, China. Soil samples from the upper 20 cm layer were collected in the field using a grid sampling method with a soil auger at 10 randomly selected points. The soil collected from each sampling point was homogenized and pooled to form a composite sample. The soil samples were transported to the laboratory in iceboxes, passed through a 60-mesh sieve to remove debris and large stones, and then stored at 4 °C until further use. For each sample, 1 g of the processed soil was mixed thoroughly with 1 mL of sterile water, allowed to stand for 30 min, and centrifuged at 3000 rpm for 5 min. The resulting supernatants were collected for genomic DNA extraction. The eight DNA templates thus obtained were subsequently analyzed by conventional PCR–AGE, dpRCA–AGE, and dpRCA–LFA in parallel. Each treatment was performed in triplicate. Given the limited number of field samples, the results are presented as preliminary applicability evidence.

## 3 Results

### 3.1 Detection principle of dpRCA

Compared with conventional RCA, the proposed dpRCA splits the conventional single PLP into two distinct oligonucleotides: LP and SP (Figure 2). Both probes are phosphorylated at their 5′ termini. The terminal regions of the LP and the entire SP are designed to be continuously complementary to adjacent sequences on the target gene. Upon simultaneous hybridization of the LP termini and the SP to the target template, the nicks are sealed by ligase, generating an intact circular template, which subsequently initiates amplification in the presence of polymerase and primers. This design ensures that a complete circular structure can be formed only when the template contains all three complementary regions corresponding to both termini of the LP and the full-length SP, thereby substantially enhancing the assay specificity. Overall, compared to traditional RCA, the present dpRCA exhibits markedly improved specificity in the formation of the circular template, offering a more stringent recognition mechanism.

**Figure 2:**
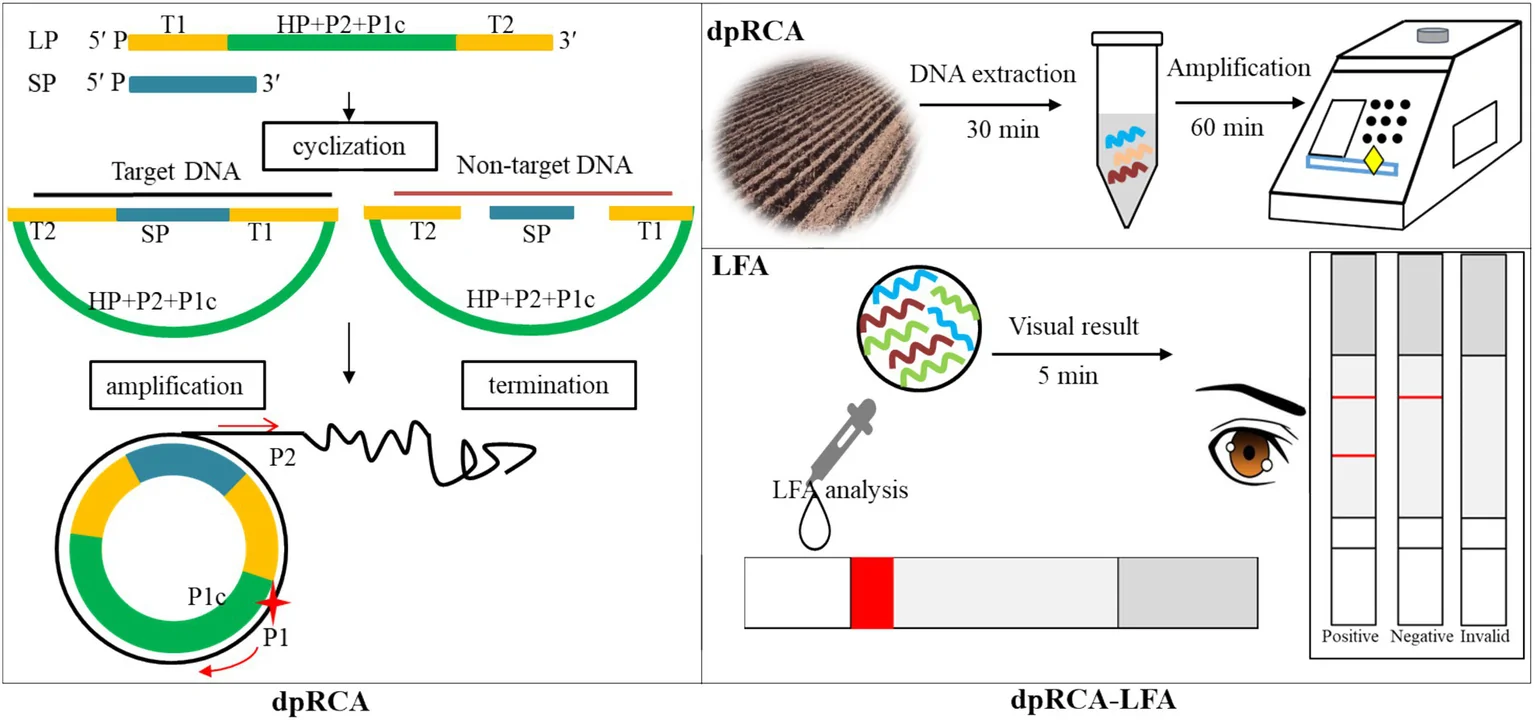
Schematic illustration of the dpRCA and dpRCA–LFA.

### 3.2 Specificity of PCR–AGE, dpRCA–AGE, and dpRCA–LFA

The specificity test results for PCR–AGE, RCA–AGE, dpRCA–AGE, and dpRCA–LFA are presented in Figure 3. PCR–AGE successfully amplified target fragments of approximately 500–700 bp from the genomic DNA of all 19 tested fungal species, including *F. proliferatum*, whereas no amplification bands were observed in the negative control, confirming the integrity and quality of the extracted DNA templates for subsequent assays (Figure 3A). In the conventional RCA–AGE analysis (Figure 3B), a positive RCA product was observed exclusively in *F. proliferatum*, while two positive results (*F. fujikuroi* and *F. verticillioides*) were also detected among the remaining 18 non-target strains. No detectable amplification bands were observed in the other non-target strains or in the blank control. In the dpRCA–AGE analysis (Figure 3C), positive dpRCA products were exclusively obtained from *F. proliferatum*, while the other 18 non-target strains, along with the blank control, exhibited no detectable amplification bands. Consistently, the dpRCA–LFA yielded results in full agreement with those of dpRCA–AGE: only the target strain displayed both the test line and the control line as pink bands on the lateral flow strips, whereas all non-target strains and the blank control showed only the control line (Figure 3D). Collectively, these findings demonstrate that the developed dpRCA-LFA assay showed high specificity within the tested strain panel for the detection of *F. proliferatum*. The specificity observed in the tested panel should be distinguished from inclusivity across the species; broader coverage of *F. proliferatum* genotypes remains to be established.

**Figure 3:**
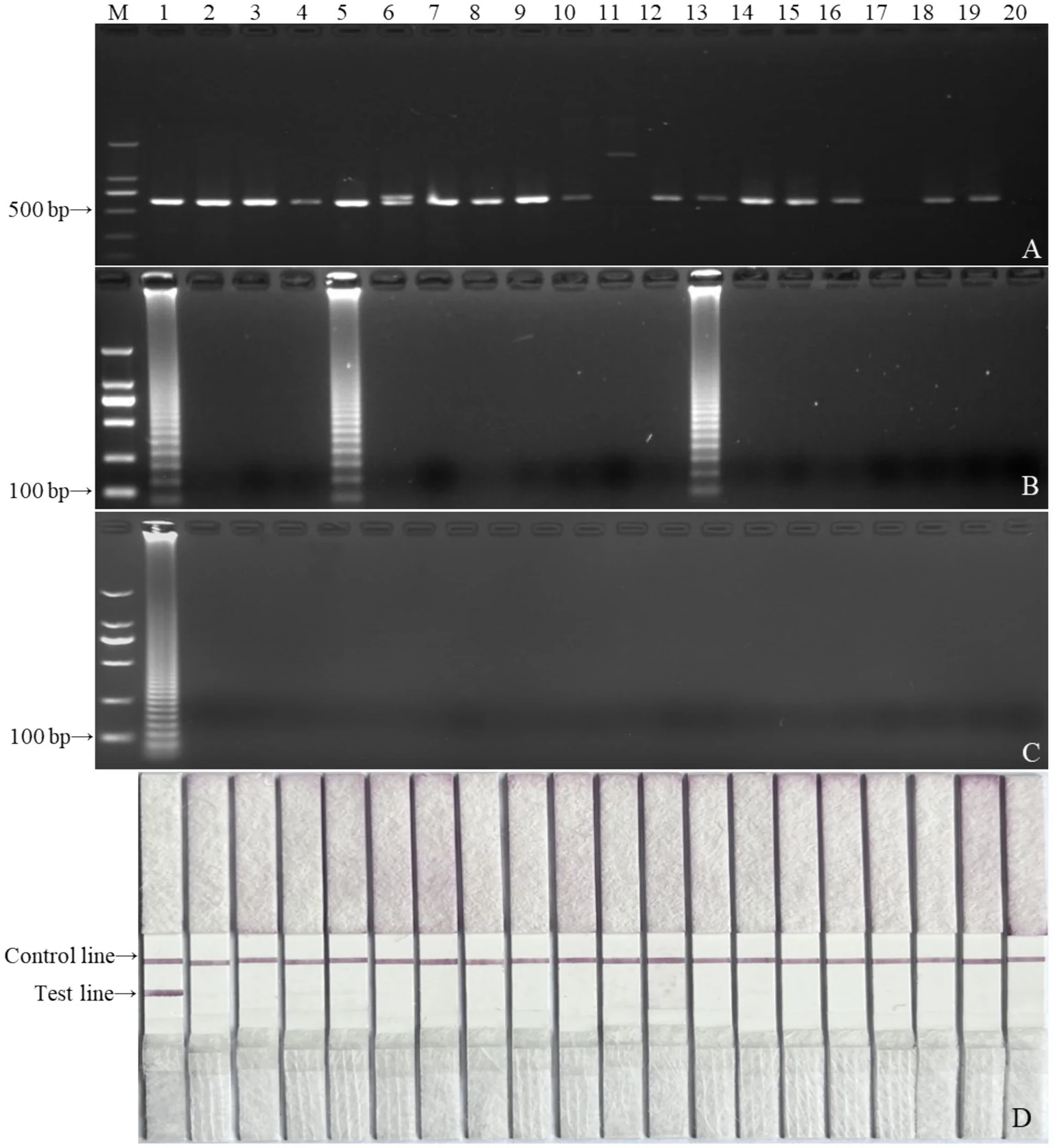
Specificity evaluation of dpRCA–LFA. **(A)** PCR–AGE. Lane M: DL 1000 DNA marker. **(B)** RCA. Lane M: DL 2000 DNA marker. **(C)** dpRCA. Lane M: DL 2000 DNA marker. **(D)** dpRCA–LFA. Lanes 1–18: *Fusarium proliferatum*, *Fusarium andiyazi*, *Fusarium asiaticum*, *Fusarium avenaceum*, *Fusarium fujikuroi*, *Fusarium boothii*, *Fusarium concentricum*, *Fusarium culmorum*, *Fusarium equiseti*, *Fusarium incarnatum*, *Fusarium graminearum*, *Fusarium meridionale*, *Fusarium verticillioides*, *Fusarium moniliforme*, *Fusarium oxysporum*, *Fusarium pseudograminearum*, *Fusarium solani*, *Fusarium temperatum*, *Fusarium tricinctum* and a blank control, respectively.

### 3.3 Sensitivity of PCR–AGE, dpRCA–AGE, and dpRCA–LFA

The analytical sensitivities of PCR–AGE, dpRCA–AGE, and dpRCA–LFA were compared under optimized conditions using genomic DNA and a recombinant plasmid harboring TEF1α as templates (Figure 4). For genomic DNA samples, conventional PCR–AGE was detected genomic DNA within the concentration range of 6.6 × 10^0^ ng μL^−1^ to 6.6 × 10^−2^ ng μL^−1^ (Figure 3A). In contrast, both dpRCA–AGE and dpRCA–LFA assays yielded positive signals across a broader range of 6.6 × 10^0^ ng μL^−1^ to 6.6 × 10^−4^ ng μL^−1^ for the same series of genomic DNA dilutions, indicating a 100-fold improvement in sensitivity over conventional PCR–AGE (Figures 3B,C). Similarly, when the recombinant plasmid DNA containing the TEF-1α gene was used as template, the positive detection range of conventional PCR–AGE was 2.7 × 10^6^ copies μL^−1^ to 2.7 × 10^4^ copies μL^−1^ (Figure 4A), whereas both dpRCA–AGE and dpRCA–LFA exhibited consistent positive detection from from 2.7 × 10^6^ copies μL^−1^ to 2.7 × 10^2^ copies μL^−1^ (Figures 4B,C). Accordingly, the LOD of conventional PCR–AGE for the recombinant plasmid (2.7 × 10^4^ copies μL^−1^) was inferior to that of dpRCA–AGE and dpRCA–LFA (2.7 × 10^2^ copies μL^−1^). Overall, the results from both template systems confirmed that dpRCA–AGE and dpRCA-LFA achieved a 100-fold improvement in detection end-point compared with conventional PCR–AGE under the tested conditions.

**Figure 4:**
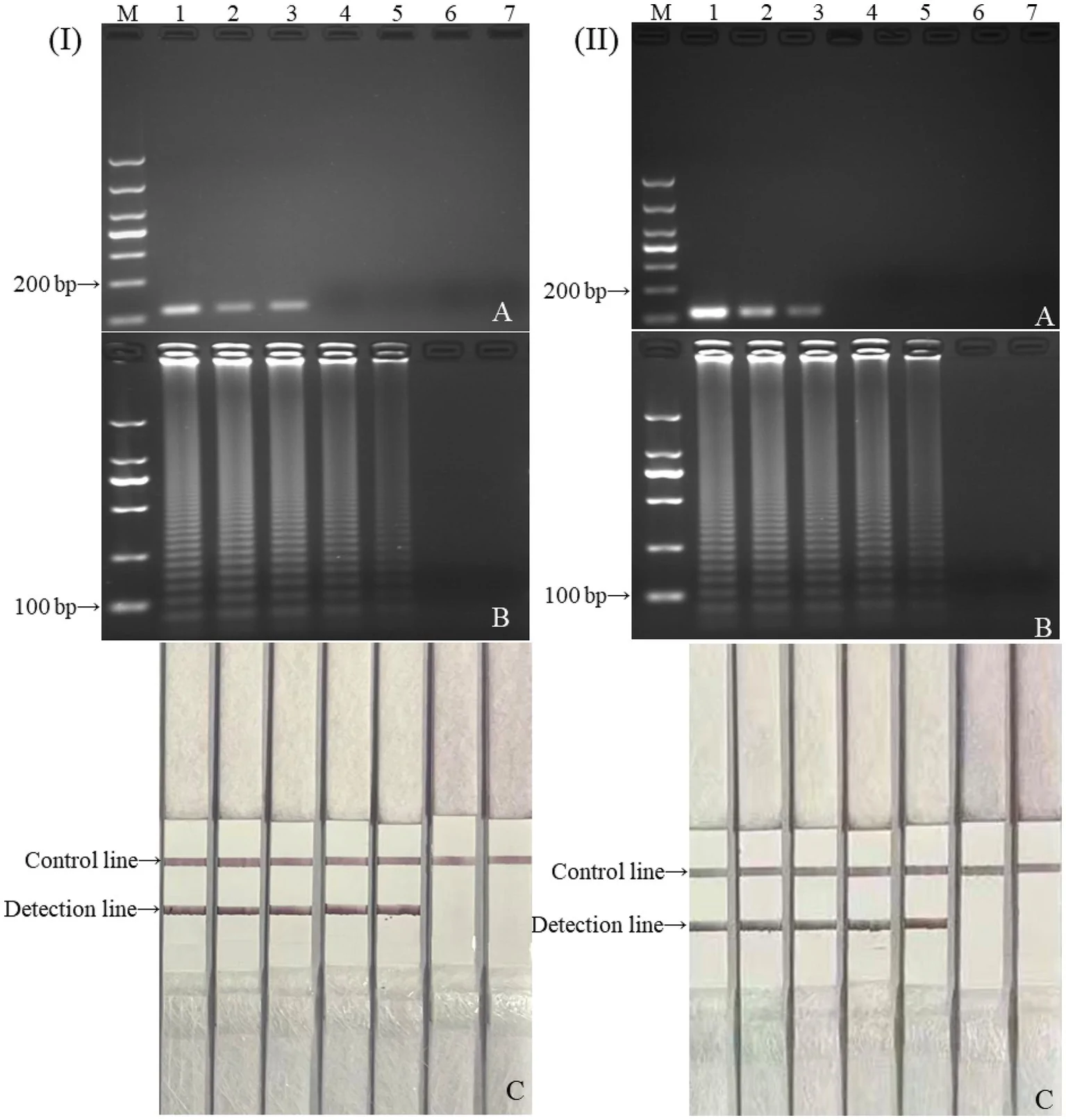
Sensitivity test of dpRCA–LFA.(I) Genomic DNA. **(A)** PCR–AGE. Lane M: DL 1000 DNA marker. **(B)** dpRCA. Lane M: DL 2000 DNA marker. **(C)** dpRCA–LFA. Lanes 1–7: 6.6 × 10^0^ ng μL^−1^, 6.6 × 10^−1^ ng μL^−1^, 6.6 × 10^−2^ ng μL^−1^, 6.6 × 10^−3^ ng μL^−1^, 6.6 × 10^−4^ ng μL^−1^, 6.6 × 10^−5^ ng μL^−1^, and blank control, respectively.(II) Recombinant Plasmid.(A) PCR–AGE. Lane M: DL 1000 DNA marker.(B) dpRCA. Lane M: DL 2000 DNA marker.(C) dpRCA–LFA. Lanes 1–7: 2.7 × 10^6^ copies μL^−1^, 2.7 × 10^5^ copies μL^−1^, 2.7 × 10^4^ copies μL^−1^, 2.7 × 10^3^ copies μL^−1^, 2.7 × 10^2^ copies μL^−1^, 2.7 × 10^1^ copies μL^−1^, and blank control, respectively.

### 3.4 Stability of PCR–AGE, dpRCA–AGE, and dpRCA–LFA

The stability of the dpRCA**–**LFA was evaluated. As shown in Figure 5A, no amplification was observed in the blank control, whereas the band intensity of PCR–AGE products gradually diminished with decreasing proportions of target genomic DNA (*F. proliferatum*) in the mixed templates. In contrast, the dpRCA–AGE amplicons exhibited consistently clear and stable band intensities across all tested ratios, with no noticeable variation resulting from increasing amounts of non-target genomic DNA (Figure 5B). Furthermore, dpRCA–LFA revealed that, despite varying compositional ratios of *F. proliferatum* to *F. graminearum* genomic DNA, consistent positive test line results were obtained among all experimental groups, while the blank control showed only the control line without the test line (Figure 5C). These results indicate that the established dpRCA–LFA produced consistent qualitative positive/negative results in the presence of non-target genomic DNA within the tested mixtures.

**Figure 5:**
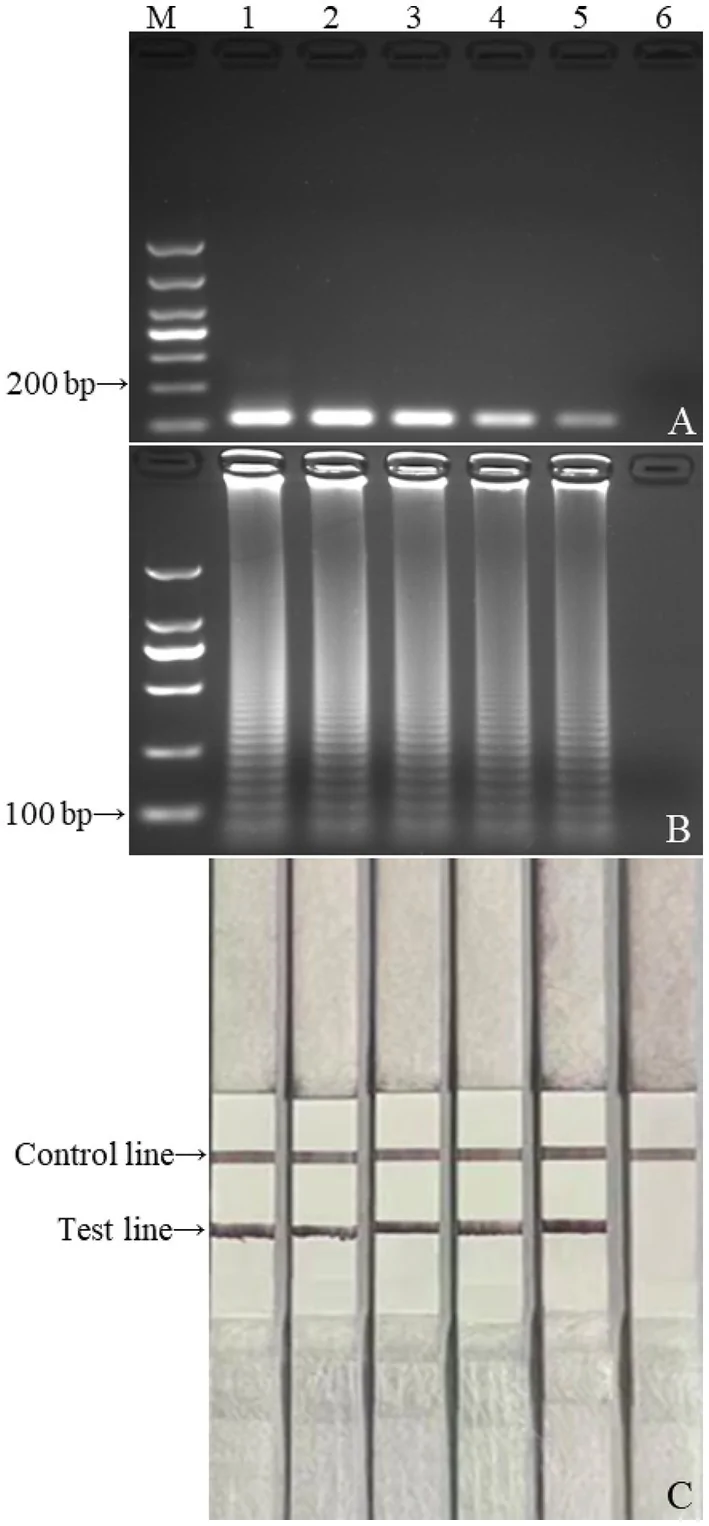
Stability evaluation of dpRCA–LFA. **(A)** PCR–AGE. Lane M: DL 1000 DNA marker. **(B)** dpRCA. Lane M: DL 2000 DNA marker. **(C)** dpRCA–LFA. Lanes 1–6: The concentration ratios of *Fusarium proliferatum* to *Fusarium graminearum* are 100:1, 10:1, 1:1, 1:10, 1:100, and blank control, respectively.

### 3.5 Detection of simulated natural samples

The DNA crude extracts obtained from simulated natural samples were analyzed using the established PCR–AGE, dpRCA–AGE, and dpRCA–LFA (Figure 6). As shown in Figure 6A, target bands were detected by conventional PCR–AGE within the DNA concentration range of 1.3 × 10^2^ ng μL^−1^ to 1.3 × 10^0^ ng μL^−1^. In contrast, positive detection was achieved by both dpRCA–AGE and dpRCA–LFA over a broader range of 1.3 × 10^2^ ng μL^−1^ to 1.3 × 10^−1^ ng μL^−1^. The band intensity of dpRCA–AGE was observed to gradually decrease with declining DNA concentration, whereas consistent positive test line results were obtained across all positive samples. Based on these results, it was concluded that the dpRCA–LFA achieves a 10-fold higher sensitivity than conventional PCR–AGE in the detection of artificially inoculated soil samples.

**Figure 6:**
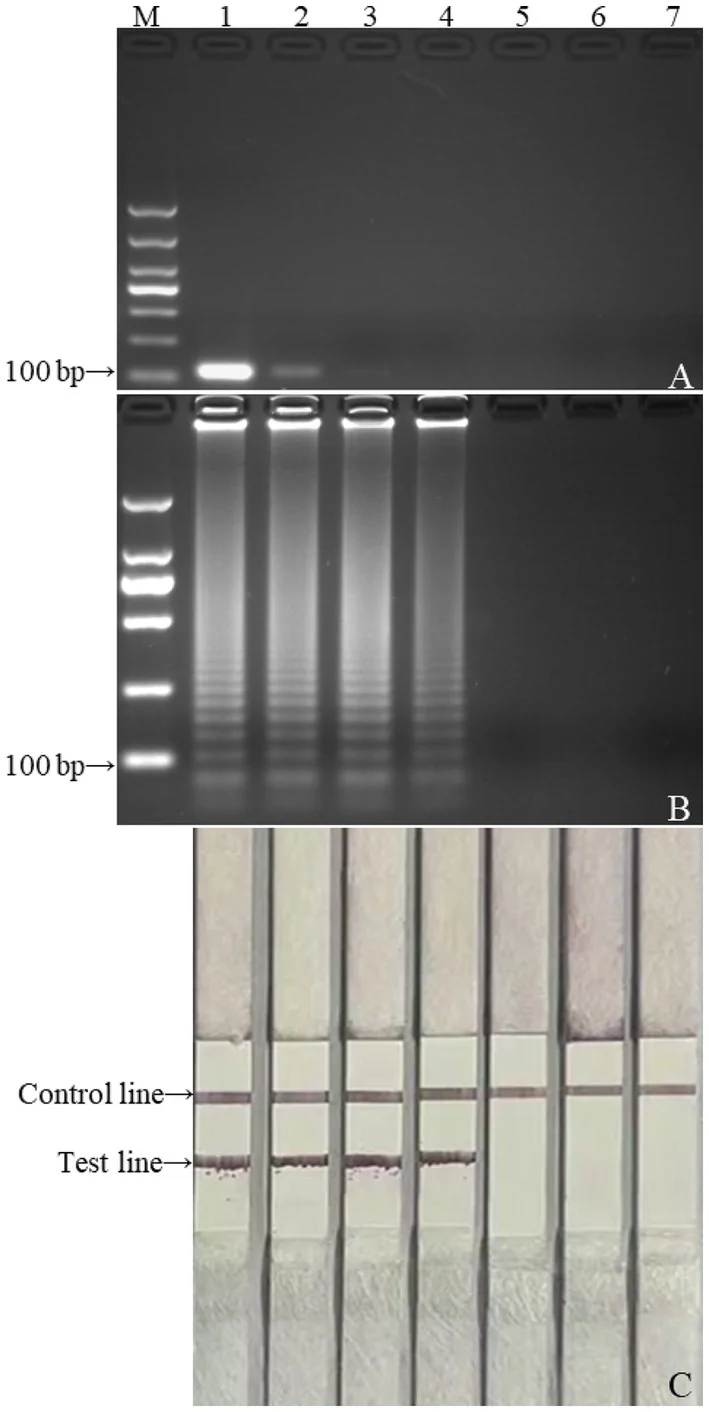
Detection of simulated natural samples using the dpRCA–LFA. **(A)** PCR–AGE; **(B)** dpRCA; **(C)** dpRCA–LFA. Lane M: DL 1000 and 2000 DNA marker; lanes 1–7: 1.3 × 10^2^ ng μL^−1^, 1.3 × 10^1^ ng μL^−1^, 1.3 × 10^0^ ng μL^−1^, 1.3 × 10^−1^ ng μL^−1^, 1.3 × 10^−2^ ng μL^−1^, 1.3 × 10^−3^ ng μL^−1^, and blank control, respectively.

### 3.6 Analysis of field samples

Eight natural soil samples were analyzed by specific PCR–AGE, dpRCA–AGE, and dpRCA–LFA using the specific primers FpF/FpR (Figure 7). Positive results were obtained for only four samples (Nos. 1, 2, 3, and 5) by specific PCR–AGE, indicating potential contamination by the target pathogen in these field samples (Figure 7A). By contrast, five samples (Nos. 1, 2, 3, 5, and 6) were tested positive by both dpRCA–AGE and dpRCA–LFA (Figures 7B,C). Notably, the band intensities of dpRCA–AGE were observed to vary among positive samples, suggesting a strong dependence on target DNA abundance, whereas consistent positive test line results were obtained by dpRCA–LFA across all positive samples. Moreover, all samples that were positive by specific PCR–AGE were also included among those detected by dpRCA–AGE and dpRCA–LFA. These findings demonstrate that dpRCA–LFA outperforms specific PCR–AGE in sensitivity and surpasses dpRCA–AGE in stability. Therefore, the established dpRCA–LFA is recommended as a more effective method for detecting low-abundance targets in practical applications. Within this limited field sample, dpRCA-LFA detected one additional positive sample compared with specific PCR-AGE. This difference is presented as a preliminary finding. Given the small sample size, larger-scale field validation across diverse geographic regions and soil types will be required before any claim of superior field performance can be made.

**Figure 7:**
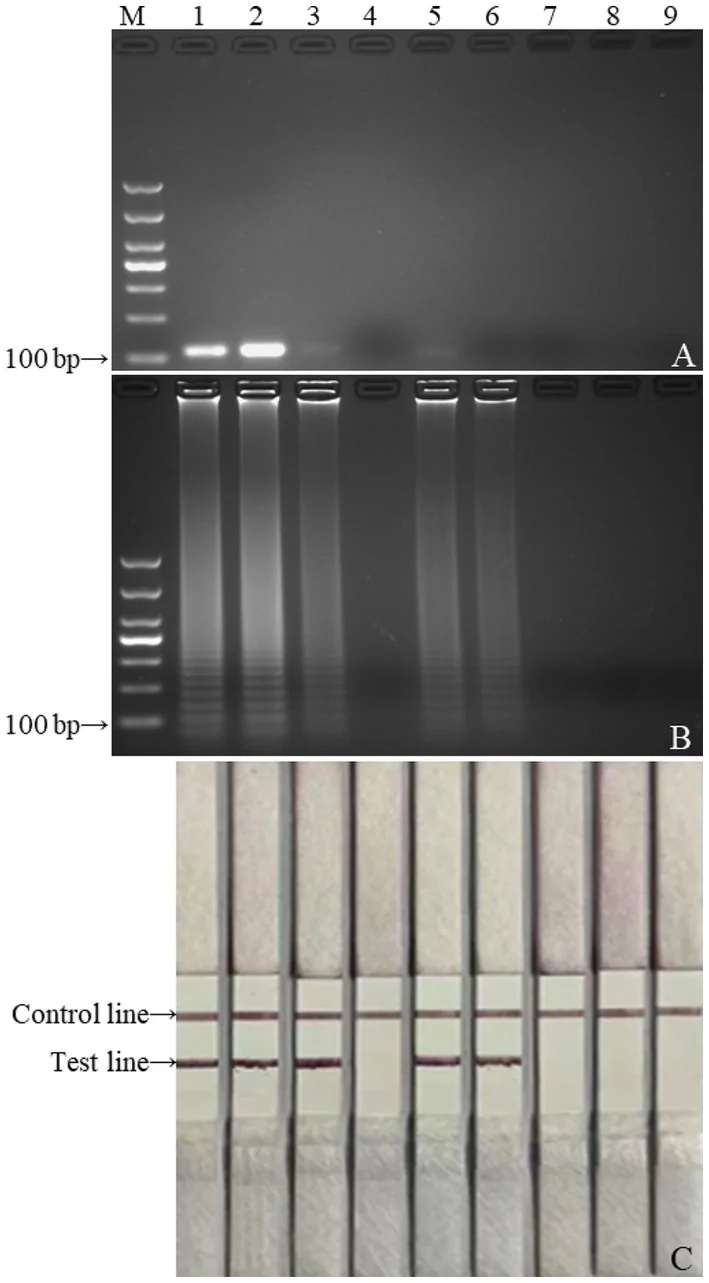
Detection of natural samples using the eRCA–LFA. **(A)** PCR–AGE. Lane M: DL 1000 DNA marker. **(B)** dpRCA. Lane M: DL 2000 DNA marker. **(C)** dpRCA–LFA. Lanes 1–9: Sample 1, Sample 2, Sample 3, Sample 4, Sample 5, Sample 6, Sample 7, Sample 8, and blank control, respectively.

## 4 Discussion

*F. proliferatum*, as an important soilborne pathogenic fungus, has become one of the major threats global crop production security due to its broad host range, strong toxigenic capacity, and wide geographical distribution. The establishment of a rapid, sensitive, and specific detection method is of great significance for early warning and precise management of the diseases caused by the pathogen. In this study, a detection assay designated as dpRCA-LFA was adapted and optimized for *F. proliferatum* by integrating dpRCA with LFA, building on previously reported dpRCA-LFA principles. The performance of this method, including its specificity, sensitivity, anti-interference capability, and practical feasibility, was systematically assessed.

The establishment of the dpRCA–LFA assay necessitates precise optimization of reaction conditions to ensure robust and reproducible detection outcomes. Among the variables investigated, probe concentration, ligation and amplification temperatures, and ligation and amplification times were found to substantially influence the overall assay performance. The following mechanistic interpretations are proposed to explain the observed optimization trends and are consistent with previous reports; however, the individual molecular contributions of hybridization, ligation, and amplification were not resolved in this study. Mechanistically, supra-optimal probe concentrations tend to promote nonspecific self-ligation through enhanced molecular collisions, thereby generating false-positive signals and highlighting a concentration-dependent competitive interplay between the probe and the target sequence (Zhang et al., 2019). Moreover, the enzymatic activities of ligase and polymerase are intrinsically thermosensitive; deviations from their respective temperature optima may induce conformational alterations or irreversible inactivation, consequently impairing probe circularization efficiency and downstream amplification yield, with a resultant loss of sensitivity (Miličić et al., 2022). Reaction time also demands meticulous adjustment—insufficient time compromises reaction completeness and signal output, whereas excessive time not only diminishes operational efficiency but also predisposes the system to nonspecific amplification products (Sun et al., 2019). Therefore, the optimization process fundamentally entails a delicate balance among assay specificity, amplification efficiency, and LOD, which holds important methodological significance for establishing standardized protocols suitable for rapid on-site detection.

A major challenge in PLP-based detection is that conventional probes may still undergo detectable ligation even when a single-base mismatch exists between the target sequence and the probe termini, thereby generating nonspecific signals and compromising assay reliability. To overcome this limitation, a dual-level specificity enhancement strategy was developed in this study, addressing both the amplification and detection phases. In the amplification step, the conventional single PLP was rationally redesigned into a bipartite system composed of a long probe and a short probe, which collectively constitute a dual-recognition mechanism (Tian et al., 2025). This dual-recognition mechanism introduces a stringent verification step that minimizes false-positive signals caused by mismatches. In the detection step, a specific oligonucleotide probe was introduced following amplification to further corroborate the identity of the amplicons via hybridization, thereby reinforcing the specificity of signal readout. This dual-layered design ensures stringent specificity control at both the initiation of dpRCA and the final signal detection. The assay specificity was evaluated against 19 fungal strains, including closely related Fusarium species, and only *F. proliferatum* yielded positive signals. The specificity observed in the tested panel should be distinguished from inclusivity across the species; broader coverage of *F. proliferatum* genotypes remains to be established. These findings confirm that the dual-recognition design, coupled with hybridization-based detection, confers high specificity and robustness, making it a promising tool for accurate pathogen diagnosis in complex sample matrices.

RCA was chosen as the primary amplification strategy in this study, given that higher pathogen concentrations are closely correlated with more rapid disease development and greater crop damage. Thus, achieving high sensitivity while maintaining rapid detection is essential for field-applicable diagnostic tools. Several molecular assays have been previously reported for the detection of *F. proliferatum*, including qPCR (5 pg. μL^−1^) (Scauflaire et al., 2012), LAMP (10 pg. μL^−1^) (Wang et al., 2020), RPA-CRISPR/Cas12a (110 pg. μL^−1^) (Jiang et al., 2025), and multiplex PCR (0.1 pg. μL^−1^) (Deng et al., 2024). The dpRCA-LFA assay developed in this study showed a lowest detected concentration of 6.6 × 10^−4^ ng μL^−1^ (0.66 pg. μL^−1^) under the tested conditions, which is comparable to the levels reported for these methods. However, comparisons across publications require caution, because reported limits may use different targets, DNA inputs, sample matrices, extraction procedures, and definitions of positivity. In addition, qPCR can support quantitative measurement when appropriately calibrated, whereas quantitative use of LAMP or RPA depends on the particular assay configuration; the present visual dpRCA-LFA readout is qualitative and does not establish a dynamic range for quantification. Nevertheless, the broader application of these techniques in field settings is restricted by their respective drawbacks: qPCR requires precise thermal cycling instruments; LAMP and RPA-CRISPR/Cas12a depend on specialized primers or reagents; and multiplex PCR relies on post-amplification electrophoresis for signal readout (Qin et al., 2019). By contrast, dpRCA-LFA features a straightforward procedure, instrument-free readout, and visible interpretation of test results, giving it potential utility for decentralized screening after suitable DNA extraction and controlled reaction processing.

The superior sensitivity of dpRCA is attributable to its dual-stage amplification mechanism, encompassing both probe circularization and rolling-circle replication (Zhou et al., 2020). Prior to amplification, the requirement for both ends of the padlock probe to perfectly match the target sequence introduces a built-in verification step that effectively reduces nonspecific background and enhances the signal-to-noise ratio (Qin et al., 2019). Following circularization, the probe is continuously elongated by a strand-displacement DNA polymerase, generating long tandem-repeat products from a single circular template through thousands of replication cycles (Liu et al., 2019). In the tested dilution series, the dpRCA-LFA assay showed a detection end-point approximately 100-fold lower than that of conventional PCR-AGE. Furthermore, although dpRCA-LFA and dpRCA-AGE showed equivalent sensitivity, the LFA format permitted result readout within 5–10 min, substantially outperforming agarose gel electrophoresis in terms of time efficiency. This feature renders the developed assay particularly attractive for rapid diagnosis at primary testing stations and in field environments.

The anti-interference performance of the developed assay further confirmed its robustness within the tested mixtures. Consistent positive results Positive signals were consistently obtained by dpRCA-LFA even when the target DNA accounted for as little as 1% of the total DNA in the presence of interfering strains. Because the readout is qualitative, these data do not establish quantitative signal stability. This stability is likely attributable to the stringent target recognition required during the circularization step, which restricts the interference of background DNA with amplification efficiency—a feature of particular relevance for environmental samples that typically harbor abundant non-target microbial DNA.

The practical utility of the method was corroborated by analyses of simulated natural samples and field samples. In artificially inoculated soil samples, dpRCA-LFA achieved a 10-fold improvement in sensitivity over conventional PCR-AGE. Although this enhancement was lower than the 100-fold increase observed in pure culture systems, it remains appreciable given the presence of reaction inhibitors (e.g., humic substances and metal ions) and the complex microbial background in soil. In field sample analysis, dpRCA-LFA identified five positive samples compared with four detected by conventional PCR-AGE. Given the limited number of field samples, this difference is presented as a preliminary observation rather than a demonstration of superior. Furthermore, the test line intensities of dpRCA-LFA were consistently uniform across positive samples, whereas the band intensities of dpRCA-AGE varied considerably, suggesting that dpRCA-LFA not only provides higher sensitivity but also offers more objective and reliable readout, although this does not constitute a quantitative measure of signal stability.

The dpRCA-LFA assay developed here is best viewed as a rapid, equipment-light screening tool for field surveillance of *F. proliferatum*. Its main advantages are a simple visual readout, minimal instrumentation requirements, and suitability for on-site testing. It is not intended to replace quantitative laboratory-based methods such as qPCR, LAMP, or RPA. Rather, dpRCA-LFA is complementary to these approaches: it can be used as a first-line screening tool in the field. Several limitations should be considered when interpreting these results. Evaluation was confined to fungal cultures and the tested soil samples; clinical applicability remains unvalidated. LFA results were interpreted qualitatively, without a validated intensity threshold or formal inter-observer analysis. Although the lateral-flow endpoint simplifies readout, the full procedure still requires sample processing, controlled reaction temperatures, contamination precautions, and trained handling.

## 5 Conclusion

In this study, a dpRCA–LFA assay was successfully developed for the rapid and specific detection of *F. proliferatum*. The dual-probe recognition mechanism improved assay discrimination within the tested strain panel. In the tested dilution series, the method showed a 100-fold improvement in detection end-point over conventional PCR-AGE, with lowest detected concentrations of 6.6 × 10^−4^ ng μL^−1^ for genomic DNA and 2.7 × 10^2^ copies μL^−1^ for recombinant plasmid containing the TEF-1α. Excellent specificity was confirmed against 19 fungal strains, and consistent positive results were maintained even when target DNA comprised only 1% of the total DNA. In soil and field samples, dpRCA-LFA detected low-abundance *F. proliferatum* DNA, identifying five positive field samples versus four by PCR-AGE; given the limited field sample size, this is a preliminary observation. The entire procedure can be completed within 90 min with visual readout in 5–10 min, requiring no specialized equipment. Nevertheless, several limitations should be acknowledged: clinical applicability remains unvalidated, LFA interpretation was qualitative. Overall, this assay offers a practical tool for detection of *F. proliferatum* in fungal cultures and soil samples within the scope tested.

## Data Availability

The raw data supporting the conclusions of this article will be made available by the authors, without undue reservation.

## Footnotes

1
https://www.ncbi.nlm.nih.gov/

2
https://www.unafold.org/mfold/applications/dna-folding-form.php
